# Diagnostic Accuracy of Body Mass Index, Bioelectrical Impedance Analysis, and Waist-to-Hip Ratio Against DEXA for Obesity in Saudi Arabia: A Cross-Sectional Study

**DOI:** 10.3390/diagnostics16152457

**Published:** 2026-08-04

**Authors:** Haifa F. Alotaibi, Saleh A. Alqahtani, Alanoud Alfraidi, Jamala Selan, Feras Altunusi, Abdulrahman Albatly, Shadan AlMuhaidib, Hanan Taib, Amr Arafat, Waleed Alhazzani

**Affiliations:** 1 Research and Innovation Institute, Ministry of Defense Health Services, Riyadh 12426, Saudi Arabia; a.alfraidi@modhs.med.sa (A.A.); ksu.jss@gmail.com (J.S.); fe.altunusi@modhs.med.sa (F.A.); s.almuhaidib@modhs.med.sa (S.A.); amr.arafat@med.tanta.edu.eg (A.A.); w.alhazzani@modhs.med.sa (W.A.); 2 Liver, Digestive, and Lifestyle Health Research Section, King Faisal Specialist Hospital and Research Center, Riyadh 12713, Saudi Arabia; 3 Organ Transplant Center of Excellence, King Faisal Specialist Hospital and Research Center, Riyadh 12713, Saudi Arabia; 4 Division of Gastroenterology & Hepatology, Weill Cornell Medicine, New York, NY 10065, USA; 5 Department of Nuclear Medicine, Prince Sultan Military Medical City, Ministry of Defense Health Services, Riyadh 12426, Saudi Arabia; dr.aalbatly@gmail.com; 6 Department of Family and Community Medicine, Prince Sultan Military Medical City, Ministry of Defense Health Services, Riyadh 12426, Saudi Arabia; hanan.altaib@gmail.com; 7 Prince Sultan Cardiac Center, Ministry of Defense Health Services, Riyadh 12426, Saudi Arabia; 8 Critical Care and Internal Medicine Department, College of Medicine, Imam Abdulrahman Bin Faisal University, Dammam 31441, Saudi Arabia

**Keywords:** obesity, dual-energy X-ray absorptiometry, bioelectrical impedance, body mass index, diagnostic accuracy

## Abstract

**Background/Objectives**: Body mass index (BMI) is the most widely used measure for obesity classification, yet it does not directly measure body fat and may substantially underestimate obesity prevalence. Simultaneous evaluation of the diagnostic accuracy of bioelectrical impedance analysis (BIA), BMI, and waist-to-hip ratio (WHR) against dual-energy X-ray absorptiometry (DEXA) for obesity classification is lacking in Saudi Arabia. We aimed to quantify the diagnostic performance of these three surrogate measures against DEXA in Saudi adults. **Methods**: We conducted a cross-sectional diagnostic accuracy study reported according to the STARD guideline at Prince Sultan Military Medical City, Riyadh, Saudi Arabia. We enrolled a convenience sample of 399 adults aged 18–65 years recruited from primary health care and outpatient clinic waiting areas. Each participant underwent InBody 770 BIA, anthropometric measurements (BMI, WHR), and DEXA (Lunar iDXA, GE) on the same day. We evaluated BIA body fat percentage against DEXA body fat percentage, BMI ≥ 30 kg/m^2^ against DEXA body fat percentage and fat mass index (FMI)-defined obesity, and WHR against the DEXA android-to-gynoid ratio. We calculated sensitivity, specificity, positive and negative predictive values, likelihood ratios, and area under the receiver operating characteristic curve (AUC), overall and stratified by sex. **Results**: DEXA classified 86.0% of participants as having obesity, compared with 62.9% by BIA and 24.8% by BMI; against DEXA body fat percentage, BMI ≥ 30 kg/m^2^ had a sensitivity of 28.9% and specificity of 100%. When FMI replaced body fat percentage as the reference standard, BMI sensitivity increased to 68.5% (specificity 97.3%, AUC 0.95). BIA achieved a sensitivity of 72.3%, specificity of 94.4%, and AUC of 0.86. WHR had the lowest sensitivity for central obesity (54.0%, specificity 94.8%, AUC 0.80). BIA showed the strongest correlation with DEXA body fat percentage (Spearman ρ = 0.82), followed by BMI (ρ = 0.60). **Conclusions**: BMI alone missed two-thirds of participants with DEXA-defined obesity in this Saudi adult cohort. Although BIA was more sensitive, it still missed 27.7% of individuals with DEXA-defined obesity. These findings support integrating body composition assessment into clinical practice and suggest that national obesity prevalence estimates based on BMI alone substantially underestimate the true burden.

## 1. Introduction

The World Health Organization defines obesity as abnormal or excessive fat accumulation that presents a risk to health [1]. In practice, clinicians and epidemiological surveys rely on body mass index (BMI) to classify obesity, using a threshold of ≥30 kg/m^2^ [2]. BMI, however, does not measure body fat directly. It cannot distinguish fat mass from lean mass, does not account for fat distribution, and varies in accuracy across sex, age, and ethnicity [3,4]. In 2025, The Lancet Diabetes and Endocrinology Commission on Clinical Obesity formalized this concern by proposing a distinction between preclinical obesity (excess adiposity with preserved tissue and organ function) and clinical obesity (excess adiposity with objective evidence of obesity-induced organ/tissue dysfunction or substantial limitations in daily activities). The Commission recommends that obesity status be confirmed using direct body fat measurement or at least one anthropometric criterion, such as waist circumference, waist-to-hip ratio (WHR), in addition to BMI, rather than relying on BMI alone [5].

In Saudi Arabia, the 2019 Saudi Arabia World Health Survey, implemented by the Ministry of Health, reported a national adult obesity prevalence of 20.2%, based on BMI-defined obesity using measured anthropometric data [6]. A recent systematic review of Saudi studies published between 2012 and 2022 confirmed this burden, reporting adult obesity prevalence ranging from 20% to 39%, with the most frequently associated comorbidities being hypertension, type 2 diabetes, and hypercholesterolemia, and an estimated national economic burden of 6.4 billion USD for obesity treatment and management [7]. These estimates, however, rely entirely on BMI-based classification and reflect its known limitations. Studies in other populations have demonstrated that BMI-based classification misses a substantial proportion of individuals with excess body fat, including those with normal BMI, a phenomenon termed normal-weight obesity [8,9]. Individuals with normal-weight obesity carry elevated cardiometabolic risk despite a BMI below 25 kg/m^2^ [8]. Whether normal-weight obesity exists at scale in the Saudi population remains uncharacterized, and, to our knowledge, the magnitude of BMI-based underdiagnosis against a reference standard remains incompletely defined in this clinical setting.

Several methods exist for assessing body composition beyond BMI. WHR reflects central adiposity but does not directly measure body fat and shows variable accuracy across populations [10,11]. Bioelectrical impedance analysis (BIA) estimates body fat percentage by measuring tissue resistance to electrical current. It offers portability and convenience, but its accuracy can be influenced by hydration status, recent physical activity, and device-specific calibration [12,13]. Dual-energy X-ray absorptiometry (DEXA) provides a comprehensive assessment of total and regional body composition, including fat mass, lean mass, and bone mineral content, and serves as a widely used reference standard for body composition analysis [14]. DEXA also enables calculation of derived indices such as fat mass index (FMI), defined as fat mass in kilograms divided by height in meters squared, which adjusts for body size and provides a more specific adiposity measure than total body fat percentage alone [15]. Additionally, DEXA quantifies regional fat distribution, including the android-to-gynoid fat ratio, a measure of central versus peripheral fat deposition [16].

Previous studies have compared BIA with DEXA for sarcopenia diagnosis and segmental fat analysis [17,18], and a limited number of studies have examined the accuracy of BMI against DEXA in Saudi adults [19,20]. However, to our knowledge, no study in Saudi Arabia has simultaneously evaluated the diagnostic accuracy of BIA-derived body fat percentage, BMI, and WHR against DEXA for obesity and central obesity classification, overall and stratified by sex, while also describing classification patterns across BMI categories. Furthermore, it remains unclear whether FMI, as a DEXA-derived size-adjusted adiposity index, changes the diagnostic performance profile of BMI compared to total body fat percentage as the reference standard.

We therefore aimed to quantify the diagnostic accuracy of three surrogate measures of adiposity, BIA-derived body fat percentage, BMI, and WHR, against DEXA in Saudi adults, overall and stratified by sex.

## 2. Objectives

We designed this study to address three primary objectives. First, we assessed the diagnostic accuracy of BIA estimates of total body fat percentage for classifying obesity against DEXA-derived total body fat percentage as the reference standard, overall and stratified by sex. Second, we evaluated the diagnostic accuracy of BMI ≥ 30 kg/m^2^ for classifying obesity against two DEXA-derived reference standards: total body fat percentage and FMI-defined obesity. Third, we assessed the diagnostic accuracy of WHR for classifying central obesity against the DEXA-derived android-to-gynoid fat ratio. As secondary objectives, we evaluated the correlation between surrogate measures (BMI, BIA estimated body fat percentage, FMI) and DEXA body fat percentage, and between WHR and the DEXA android-to-gynoid ratio.

## 3. Methods

### 3.1. Study Design

We conducted a comparative cross-sectional diagnostic accuracy study reported according to the Standards for Reporting Diagnostic Accuracy Studies (STARD) guideline [21]. A completed STARD checklist is provided in Supplementary Table S1. We compared body composition measurements obtained from BIA and anthropometric indices (BMI, WHR) with those from DEXA-derived body composition measures, which served as the reference standard.

### 3.2. Ethical Considerations

All participants provided written informed consent before enrollment. We collected and stored data securely using REDCap (Research Electronic Data Capture, version 13.7.9; Vanderbilt University, Nashville, TN, USA) software, with no personal identifiers recorded. The study was conducted in accordance with the Declaration of Helsinki, and the Central Ethics Review Board at the Ministry of Defense Health Services approved the study (protocol code 11-2025-02-03-108, approved 27 April 2025).

### 3.3. Setting and Participants

We recruited adult participants aged 18–65 years from the waiting area of the primary health care clinic and outpatient clinics at Prince Sultan Military Medical City, Riyadh, Saudi Arabia, between 14 October 2025 and 17 February 2026. A research coordinator approached potential participants, provided study information, and obtained written informed consent. We excluded pregnant women, individuals with metal implants, those exceeding the DEXA scanner’s weight of 204 kg or width limits, and those unable to lie supine and still for the scan duration. Recruitment followed a convenience sampling strategy.

### 3.4. Sample Size

We estimated the required sample size using Buderer’s formula for diagnostic accuracy studies. We applied 90% expected sensitivity, 85% expected specificity, 20% prevalence, 0.07 precision, and a 95% confidence level. We based the expected diagnostic performance on a meta-analysis of BIA against DEXA [22]. We used a prevalence of 20%, consistent with the 2019 World Health Survey estimate of BMI-defined adult obesity (BMI ≥ 30 kg/m^2^) and within the range reported in a recent systematic review of adult obesity prevalence (20–39%) in Saudi Arabia [6,7]. The sensitivity-based calculation yielded a minimum of 353 participants. After adjusting for an anticipated 10% dropout rate, the target enrollment was 393.

### 3.5. Measurements

Each participant underwent all measurements in a single day. We collected demographic data (age, sex, nationality, occupation, marital status, smoking status, physical activity status) and self-reported comorbidity information (hypertension, diabetes, dyslipidaemia, cardiovascular disease, stroke, renal disease, and other conditions). A research coordinator documented all measurements using a standardized data collection sheet in REDCap.

We measured height to the nearest 0.1 cm using a wall-mounted stadiometer and weight to the nearest 0.1 kg using a calibrated digital scale, with participants wearing light clothing and no shoes. We calculated BMI as weight in kilograms divided by height in meters squared. We measured waist circumference at the midpoint between the lower rib margin and iliac crest using a non-stretchable tape measure, and hip circumference at the widest point of the buttocks. We calculated WHR as waist circumference divided by hip circumference. We measured blood pressure using a GE B20 automatic non-invasive blood pressure monitor after 5–7 min of seated rest, recording systolic and diastolic values in mmHg.

We assessed body composition using the InBody 770 (InBody Co., Ltd., Seoul, Republic of Korea), a multi-frequency bioelectrical impedance device operating at 1, 5, 50, 250, 500, and 1000 kHz across eight tactile electrodes. Participants stood barefoot on the platform and grasped the hand electrodes for the duration of the measurement. The device recorded total body fat percentage, skeletal muscle mass, visceral fat area, and segmental lean and fat analysis. We recorded fasting status and time since last exercise for each participant.

A trained technician performed DEXA scans using a Lunar iDXA (GE, Healthcare, Madison, WI, USA; Software Version 17). Participants wore a patient gown and lay supine on the scanning table. We obtained measurements of total body fat percentage, total fat mass (kg), lean body mass, bone mineral density, bone mineral content, and regional body composition (fat and lean mass distribution in the arms, legs, and trunk). We calculated FMI as DEXA-derived total fat mass in kilograms divided by height in meters squared. We calculated the android-to-gynoid fat ratio from DEXA-derived regional fat distribution data as a measure of central adiposity.

Participants proceeded from one measurement to the next without a fixed time interval and with no clinical intervention between tests. The InBody 770 generated results automatically without subjective operator interpretation, and the DEXA scan was processed by standardized software; therefore, neither measurement was subject to reader interpretation. Results from the index test and reference standard were not reviewed by the respective operators at the time of measurement, as device-generated results were extracted after completion of all assessments.

### 3.6. Definitions

We defined obesity based on the test used: body fat percentage ≥ 25% in men and ≥35% in women by DEXA scan [23], body fat percentage ≥ 25% in men and ≥35% in women by BIA [24], BMI ≥ 30 kg/m^2^ [1], and FMI of >9 kg/m^2^ for men or >13 kg/m^2^ for women [25]. We defined central obesity as WHR > 0.90 for men and >0.85 for women [26], and as an android-to-gynoid fat ratio > 1.0 for men and >0.8 for women on DEXA scan. Because no DEXA-specific cutoff consensus for the android-to-gynoid ratio has been established, these sex-specific thresholds were adopted from conventional criteria for android central fat distribution [27].

Other BMI categories included ≤24.9 kg/m^2^ (normal weight /underweight), 25.0–29.9 kg/m^2^ (overweight), and ≥30.0 kg/m^2^ (obesity), consistent with WHO classification [1], whereas other FMI categories included 3–6 kg/m^2^ (normal) and >6–9 kg/m^2^ (overfat) for men, and 5–9 kg/m^2^ (normal) and >9–13 kg/m^2^ (overfat) for women [25].

### 3.7. Index Test and Reference Standard Comparisons

We structured the diagnostic accuracy analyses around four comparisons. First, we compared BIA-derived total body fat percentage (index test) with DEXA-derived total body fat percentage (reference standard) for the classification of obesity. Second, we compared BMI ≥ 30 kg/m^2^ (index test) against DEXA-derived total body fat percentage (reference standard) for the classification of obesity. Third, we compared BMI ≥ 30 kg/m^2^ (index test) against FMI-defined obesity (alternative DEXA-derived reference standard) for the classification of obesity. Fourth, we compared WHR above sex-specific thresholds (index test) with the DEXA-derived android-to-gynoid fat ratio (reference standard) for the classification of central obesity.

### 3.8. Statistical Analysis

We performed all analyses using R software (R Foundation for Statistical Computing, Vienna, Austria; version 4.5.0). We considered a two-sided *p*-value < 0.05 statistically significant.

We reported continuous variables as mean ± standard deviation if normally distributed, or as median (interquartile range) if non-normally distributed. We reported categorical variables as counts with percentages. We compared continuous variables across the three BMI groups using the Kruskal–Wallis test and categorical variables using the chi-square test (or Fisher’s exact test when expected cell counts were <5).

For each comparison, we constructed two-by-two cross-classification tables and calculated sensitivity, specificity, positive predictive value (PPV), negative predictive value (NPV), and positive and negative likelihood ratios (LRs) with 95% confidence intervals (CIs). We generated receiver operating characteristic (ROC) curves and calculated the area under the curve (AUC) to quantify overall discriminative ability. We summarized the distribution of obesity classifications based on DEXA-derived body fat percentage and FMI across WHO BMI categories. We performed all diagnostic accuracy analyses in the overall sample and stratified by sex.

We assessed the correlation between surrogate measures (BMI, BIA-estimated body fat percentage, FMI) and DEXA body fat percentage, and between WHR and the DEXA android-to-gynoid ratio, using Spearman’s rank correlation coefficients. We selected Spearman’s method because body composition variables were non-normally distributed. We performed each analysis using all participants with complete data for the relevant comparison (complete-case analysis).

## 4. Results

### 4.1. Study Population

Of 399 enrolled participants, DEXA body composition data were available for 386 (96.7%) and BIA measurements for 394 (98.7%) (Supplementary Figure S1). The cohort included 148 males (37.1%) and 251 females (62.9%), with a median age of 37 years (IQR 32–44). Participants were stratified into three BMI groups: ≤24.9 kg/m^2^ (*n* = 151; 37.8%), 25.0–29.9 (*n* = 149; 37.3%), and ≥30.0 (*n* = 99; 24.8%).

### 4.2. Participant Characteristics

Baseline demographic, anthropometric, and clinical characteristics are presented in Table 1. Weight, waist circumference, WHR, and blood pressure increased significantly across BMI strata (all *p* < 0.001). Among comorbidities, diabetes prevalence differed across groups (6% vs. 12.8% vs. 15.2%; *p* = 0.04). Hypertension showed a numerical gradient that did not reach significance (*p* = 0.11). Other comorbidities did not differ across BMI groups (Table 1).

### 4.3. Obesity Classification by Method

DEXA body fat percentage criteria classified 332 of 386 participants (86.0%) as having obesity, compared with 248 of 394 (62.9%) by BIA and 99 of 399 (24.8%) by BMI. Among participants with ≤24.9 kg/m^2^, 65.1% met DEXA criteria for obesity. In the overweight stratum (BMI: 25.0–29.9 kg/m^2^), 97.9% met the DEXA body fat percentage threshold for obesity, whereas all participants with BMI ≥ 30 kg/m^2^ met this DEXA threshold. Sex-stratified DEXA-defined obesity prevalence was similar in males and females (85.4% vs. 86.4%). Cross-classification tables for all index tests, overall and by sex, are provided in Table 2 and Supplementary Table S2.

FMI-based classification varied across BMI strata (Supplementary Table S3). Among participants with BMI ≤ 24.9 kg/m^2^, only 1.4% met FMI criteria for obesity, whereas 32.2% were classified as overfat. Among those with BMI ≥ 30 kg/m^2^, 92.7% met FMI criteria for obesity (Table 1).

### 4.4. Diagnostic Accuracy of BMI

**Figure 1:**
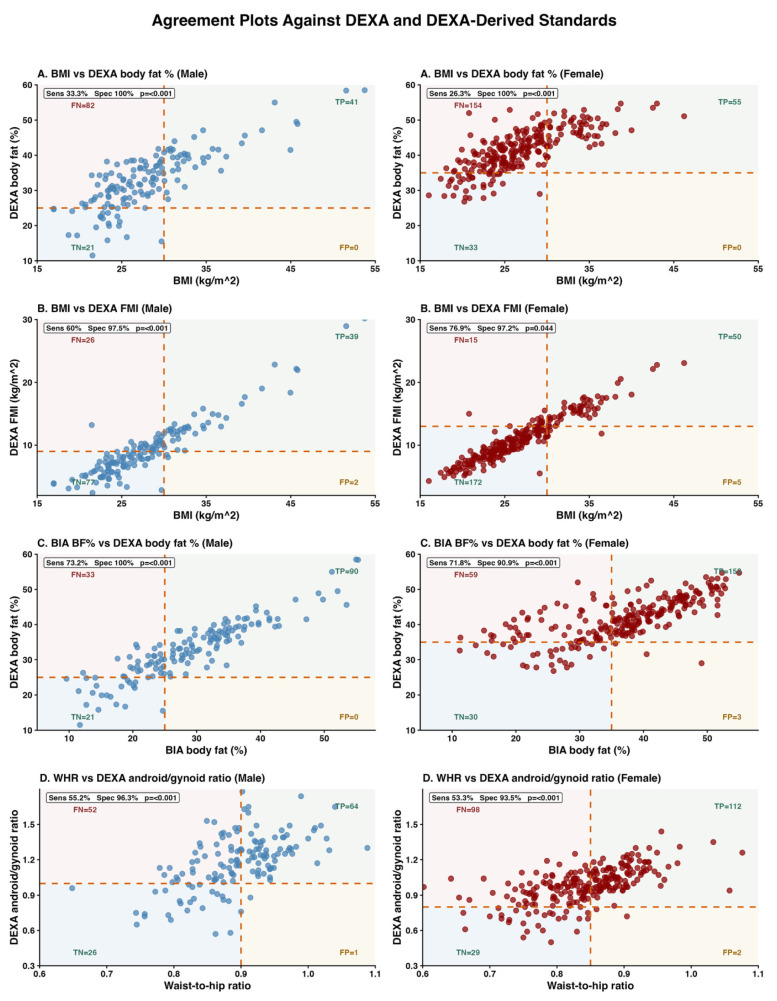
Agreement between index tests and DEXA reference standards for obesity and central obesity classification, stratified by sex. Each panel displays individual participant data plotted against the index test (x-axis) and the corresponding DEXA reference standard (y-axis). Dashed vertical lines indicate the index test threshold; dashed horizontal lines indicate the DEXA reference threshold. The intersection of threshold lines defines four diagnostic quadrants: true positive (upper right), false negative (upper left), true negative (lower left), and false positive (lower right). Quadrant counts are annotated in each panel. Sensitivity, specificity, and *p*-value from the chi-square test are displayed in the upper-left inset of each panel. Blue points represent males (left column); red points represent females (right column). Panel (**A**) BMI (kg/m^2^) versus DEXA total body fat percentage. Index test threshold: BMI ≥ 30 kg/m^2^. Reference standard threshold: body fat percentage ≥ 25% for males and ≥35% for females. Panel (**B**) BMI (kg/m^2^) versus DEXA-derived fat mass index (FMI, kg/m^2^). Index test threshold: BMI ≥ 30 kg/m^2^. Reference standard threshold: FMI > 9 kg/m^2^ for males and >13 kg/m^2^ for females. Panel (**C**) BIA body fat percentage versus DEXA total body fat percentage. Both thresholds: ≥25% for males and ≥35% for females. Panel (**D**) waist-to-hip ratio versus DEXA android-to-gynoid fat ratio. Index test threshold: WHR > 0.90 for males and >0.85 for females. Reference standard threshold: android-to-gynoid ratio >1.0 for males and >0.8 for females. The shaded region in the upper-left quadrant (false negatives) highlights participants not classified as having obesity by the index test but classified as having obesity by the DEXA reference standard. *n* = 386 for panels (**A**–**C**) (DEXA body fat percentage and FMI available), and *n* = 384 for panel (**D**) (android-to-gynoid ratio available).

When compared to DEXA body fat percentage, a BMI threshold of ≥30 kg/m^2^ yielded a sensitivity of 28.9% (95% CI 24.1–34.1), specificity of 100.0% (95% CI 93.4–100.0), PPV of 100.0% (95% CI 96.2–100.0), and NPV of 18.6% (95% CI 14.3–23.6) (Table 3); sex-stratified scatter plots with diagnostic quadrants are shown in Figure 1A.

**Figure 2:**
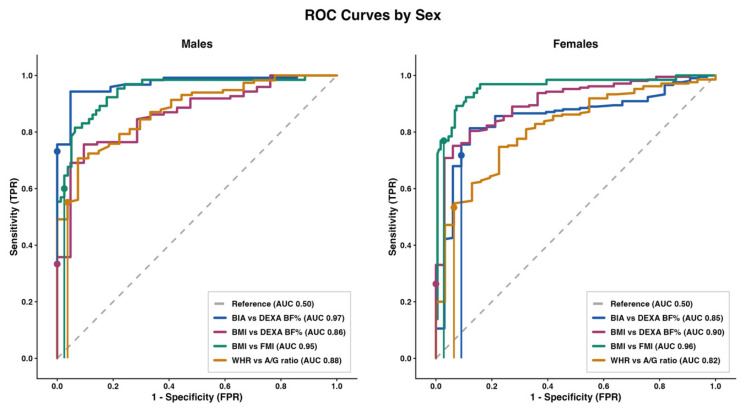
Receiver operating characteristic curves for four diagnostic comparisons, stratified by sex. Each panel displays the ROC curves for BIA body fat percentage versus DEXA body fat percentage (blue), BMI versus DEXA body fat percentage (pink), BMI versus DEXA-derived fat mass index (teal), and waist-to-hip ratio versus DEXA android-to-gynoid ratio (amber). The dashed diagonal line represents the reference line (AUC = 0.50). Filled circles on each curve indicate the operating point at the pre-specified clinical threshold. The area under the curve (AUC) for each comparison is shown in the legend. Left panel: males (*n* = 144 for BIA vs. DEXA BF%, *n* = 144 for BMI vs. DEXA BF%, *n* = 144 for BMI vs. FMI, *n* = 143 for WHR vs. A/G ratio). In males, BIA achieved the highest AUC (0.97), followed by BMI vs. FMI (0.95), WHR vs. A/G ratio (0.88), and BMI vs. DEXA BF% (0.86). Right panel: females (*n* = 242 for BIA vs. DEXA BF%, *n* = 242 for BMI vs. DEXA BF%, *n* = 242 for BMI vs. FMI, *n* = 241 for WHR vs. A/G ratio). In females, BMI vs. FMI achieved the highest AUC (0.96), followed by BMI vs. DEXA BF% (0.90), BIA (0.85), and WHR (0.82).

Continuous BMI yielded an AUC of 0.89 (95% CI 0.84–0.93) against the same reference; sex-stratified ROC curves are shown in Figure 2.

When compared to FMI, a BMI threshold of ≥30 kg/m^2^ yielded a sensitivity of 68.5% (95% CI 59.7–76.3), specificity of 97.3% (95% CI 94.4–98.9), PPV of 92.7% (95% CI 85.6–97.0), NPV of 85.9% (95% CI 81.3–89.7), +LR of 25.04, −LR of 0.32 (Table 3); sex-stratified scatter plots with diagnostic quadrants are shown in Figure 1B. Continuous BMI yielded an AUC of 0.95 (95% CI 0.92–0.97) against the same reference.

### 4.5. Diagnostic Accuracy of BIA

When compared with DEXA body fat percentage, BIA yielded a sensitivity of 72.3% (95% CI 67.1–77.0), specificity of 94.4% (84.6–98.8), PPV of 98.8% (95% CI 96.4–99.7), NPV of 35.7% (95% CI 27.8–44.1), +LR of 13.01, and −LR of 0.29 (Table 3); sex-stratified scatter plots with diagnostic quadrants are shown in Figure 1C. As a continuous measure, BIA-estimated body fat percentage yielded an AUC of 0.86 (95% CI 0.81–0.91) against the same reference; sex-stratified ROC curves are shown in Figure 2.

### 4.6. Diagnostic Accuracy of WHR for Central Obesity

When compared to android-to-gynoid ratio on DEXA, WHR yielded a sensitivity of 54.0% (95% CI 48.4–59.5), specificity of 94.8% (95% CI 85.6–98.9), PPV of 98.3% (95% CI 95.2–99.7), NPV of 26.8% (95% CI 20.9–33.4), +LR of 10.44, and −LR of 0.49 (Table 3). As a continuous measure, WHR yielded an AUC of 0.80 (95% CI 0.75–0.86) against the same reference. Sensitivity was consistently low across sexes (males 55.2%, females 53.3%).

### 4.7. Correlations with DEXA-Derived Body Composition

**Figure 3:**
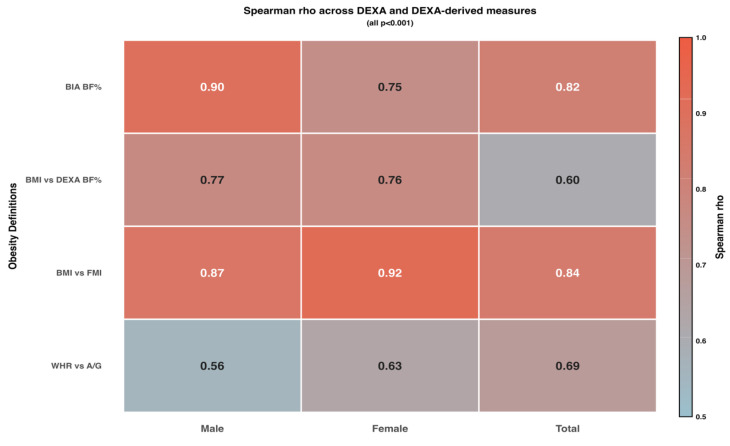
Spearman correlation coefficients between surrogate obesity measures and DEXA reference standards, stratified by sex. Heatmap displaying Spearman rank correlation coefficients (ρ) for each surrogate measure against its corresponding DEXA reference standard, stratified by male, female, and total sample. The colour scale ranges from 0.5 (teal) to 1.0 (dark red), with exact ρ values annotated within each cell. The first row (BIA BF%) and second row (BMI vs. DEXA BF%) are correlated with DEXA total body fat percentage. The third row (BMI vs. FMI) shows the correlation between BMI and the DEXA-derived fat mass index. The fourth row (WHR vs. A/G) is correlated with the DEXA android-to-gynoid fat ratio, a different reference variable, and is visually separated by a horizontal border. BIA body fat percentage demonstrated the strongest correlation with DEXA body fat percentage (ρ = 0.900 males, 0.747 females, 0.820 total), followed by BMI (ρ = 0.768 males, 0.764 females, 0.605 total). BMI correlated more strongly with FMI (ρ = 0.872 males, 0.924 females, 0.844 total) than with DEXA body fat percentage. WHR showed the weakest correlation with its DEXA reference (ρ = 0.556 males, 0.635 females, 0.686 total). All correlations were statistically significant (*p* < 0.001). Males *n* = 144 (BIA, BMI), *n* = 143 (WHR); females *n* = 242 (BIA), *n* = 241 (WHR). BF%, body fat percentage; BMI, body mass index; FMI, fat mass index; WHR, waist-to-hip ratio; A/G, android-to-gynoid ratio; DEXA, dual-energy X-ray absorptiometry.

Body fat percentage measured by BIA showed a strong correlation with DEXA measurement (Spearman rho = 0.82, *p* < 0.001) (Table 4, Figure 3). In addition, BMI showed a strong correlation with FMI (rho = 0.84) and a moderate correlation with DEXA body fat percentage (rho = 0.60). WHR showed a moderate correlation with the DEXA android-to-gynoid ratio (rho = 0.69). Sex-stratified correlations are presented in Table 4.

## 5. Discussion

This study assessed the diagnostic accuracy of BMI, BIA, and WHR against DEXA in a convenience sample of 399 adults in Saudi Arabia. DEXA classified 86% of participants as having obesity, compared with 62.9% by BIA and 24.8% by BMI. While BMI achieved a specificity of 100%, the sensitivity was very low (28.9%), missing 236 of 332 individuals with DEXA-defined obesity. BIA achieved higher sensitivity (72.3%), with specificity of 94.4%; it still missed 92 of 332 participants with DEXA-defined obesity. WHR detected only 54.0% of participants with DEXA-defined central obesity despite high specificity (94.8%). When compared to FMI, sensitivity of BMI rose from 28.9% to 68.5%, with an AUC of 0.95. Total body fat percentage measured using BIA showed the strongest correlation with DEXA measurement (Spearman rho = 0.82).

The discordance between BMI-defined and DEXA-defined obesity highlights the limitations of BMI as a standalone marker of adiposity in this population. BMI functioned as a rule-in test (specificity 100%) but demonstrated limited value as a screening tool, identifying fewer than one in three individuals with DEXA-defined obesity. About 65% of participants with BMI < 25 kg/m^2^ and 97.9% of participants with BMI 25.0–29.9 kg/m^2^ met DEXA obesity criteria, revealing extensive normal-weight and overweight-associated excess adiposity that BMI-based practice may overlook.

Although BIA showed better diagnostic accuracy than BMI, 27.7% of participants with DEXA-defined obesity were missed by BIA. Sex differences appeared primarily in specificity (males 100% vs. females 90.9%) and AUC (males 0.97 vs. females 0.85), while sensitivity was comparable (males 73.2%, females 71.8%).

When using height-standardized metrics, BMI performed better against FMI-defined obesity (sensitivity 68.5%, AUC 0.95) than against total body fat percentage (sensitivity 28.9%). Because FMI adjusts fat mass for height, this finding suggests that part of the BMI–DEXA discordance may reflect the limitation of total body fat percentage to account for body size rather than BMI insensitivity alone. FMI may represent a more appropriate DEXA-derived reference standard for evaluating BMI, as both metrics are height-normalized.

WHR detected only 54.0% of participants with elevated DEXA android-to-gynoid ratios, with consistent low sensitivity across sexes. The moderate WHR–DEXA android-to-gynoid correlation (rho = 0.69) suggests that WHR provides only a rough approximation of central fat distribution.

Our findings align directionally with international and regional evidence but show a larger magnitude of discordance between BMI-defined and DEXA-defined obesity. In a recent NHANES analysis of 10,747 US adults, a study reported BMI sensitivity of 74.6% and waist circumference sensitivity of 88.5% against DEXA-defined excess adiposity [28], while an earlier large NHANES study found that BMI ≥ 30 kg/m^2^ sensitivity was 36–49% against bioelectrical impedance-estimated body fat in 13,601 US adults [24], and another US clinical cohort reported that BMI classified only 26% of adults as having obesity versus 64% by DEXA [29]. Within Saudi Arabia, BMI ≥ 30 kg/m^2^ sensitivity was 34% in men and 55% in women against DEXA-defined obesity in 942 primary care adults, with optimal cutoffs lowered to BMI ≥ 24 kg/m^2^ [20], and BMI misclassified 40.6% of 319 Eastern Province adults [19]. A recent application of the 2025 Lancet Diabetes and Endocrinology Commission criteria to US NHANES data confirmed that combining BMI with body composition and waist-based measures substantially raises national obesity prevalence estimates above BMI alone [30]. Our overall BMI sensitivity of 28.9% sits below these comparators, reflecting the higher DEXA-obesity prevalence in our convenience sample (86%) and the higher body fat at a given BMI documented in South Asian populations [31,32] and Middle Eastern populations [33].

Current international guidelines have moved obesity diagnosis beyond BMI alone. The 2024 European Association for the Study of Obesity (EASO) framework moved obesity diagnosis beyond BMI alone and operationalized a two-component model based on anthropometric (excess adiposity) and clinical (medical, functional, or psychological impairments) diagnosis. The framework recommends waist circumference in any person with BMI < 35 kg/m^2^, a waist-to-height ratio threshold of >0.5, ethnicity-specific BMI cutoffs, and DEXA or BIA when BMI is ambiguous [34]. The 2025 Lancet Diabetes and Endocrinology Commission introduced the distinction between preclinical and clinical obesity and recommended confirming excess adiposity by direct body fat measurement or at least one additional anthropometric criterion beyond BMI [5]. In a population-based cohort of 1,472,819 adults, the type 2 diabetes risk-equivalent BMI cutoff for Arab populations was estimated at 26.6 kg/m^2^, substantially lower than the conventional WHO threshold of 30 kg/m^2^ [35]. Against this guideline backdrop, our study evaluated the diagnostic accuracy of BMI, BIA, and WHR against DEXA in Saudi adults, providing empirical evidence for which surrogate measures most reliably identify excess adiposity in this population.

### Strengths

We simultaneously evaluated three surrogate measures against DEXA in the same participants, applied sex-specific thresholds, stratified diagnostic accuracy analyses by sex, and described obesity classifications across BMI categories. The inclusion of FMI as an alternative reference standard is novel in the Saudi context and clarifies why BMI and DEXA body fat percentage disagree. The study reported according to STARD guidelines and employed a comprehensive statistical framework including ROC analysis, likelihood ratios, and correlation assessment.

## 6. Limitations

Convenience sampling from a single hospital outpatient setting limits generalizability. This sampling strategy may have selected participants with higher adiposity than the general Saudi adult population. We did not standardize fasting or hydration before InBody assessment, potentially introducing BIA measurement variability. The cross-sectional design precludes assessment of temporal stability or predictive validity. The DEXA android-to-gynoid ratio threshold requires explicit definition and validation. The FMI thresholds applied here are derived from a US reference population and, like the body fat percentage criteria, have not been validated against cardiometabolic outcomes in Arab adults. We did not collect ethnicity data beyond Saudi nationality, preventing assessment of within-population variation.

Future studies should validate these findings in population-based samples with standardized pre-measurement protocols and establish Saudi-specific body fat percentage and FMI thresholds optimized against cardiometabolic outcomes. Prospective cohorts should assess whether DEXA-defined normal-weight obesity predicts incident diabetes, cardiovascular disease, and mortality. Implementation studies should evaluate the clinical utility and cost-effectiveness of incorporating InBody BIA into routine primary care screening.

## 7. Conclusions

In this sample of Saudi adults, BMI ≥ 30 kg/m^2^ detected fewer than one-third of participants with DEXA-defined obesity, and 65% of participants with BMI < 25 kg/m^2^ met the same body fat percentage criteria. InBody BIA improved sensitivity but still missed 27.7% of individuals with DEXA-defined obesity. WHR performed poorly for central obesity screening. These findings demonstrate that BMI alone substantially underestimates obesity burden and support further evaluation of body composition assessment into clinical practice and epidemiological surveillance. The high proportion of participants classified as having obesity by DEXA reflects this convenience sample and should not be interpreted as a population prevalence estimate. Larger population-based studies using systematic sampling and multiple body composition measures are needed to define the true obesity burden.

## Figures and Tables

**Table 1:** Baseline characteristics of study participants.

Characteristics	Total (*n* = 399)	BMI ≤ 24.9 (*n* = 151 (37.8%))	BMI 25.0–29.9 (*n* = 149 (37.3%))	BMI ≥ 30.0 (*n* = 99 (24.8%))	*p*-Value
Age (years, median (IQR))	37 (32–44.8)	35 (29.2–42.8)	38 (33–44)	38 (33–47.5)	0.01
Age Group (*n*, %)					
<50 years	334 (83.9%)	126 (84%)	128 (85.9%)	80 (80.8%)	
≥50 years	64 (16.1%)	24 (16%)	21 (14.1%)	19 (19.2%)	0.56
Sex (*n*, %)					
Male	148 (37.1%)	51 (33.8%)	55 (36.9%)	42 (42.4%)	
Female	251 (62.9%)	100 (66.2%)	94 (63.1%)	57 (57.6%)	0.38
Height (cm, median (IQR))	161 (155–169)	160 (156–167)	162 (155–170)	160 (154–169.5)	0.48
Weight (kg, median (IQR))	70 (59.5–81)	57.8 (52.5–64.2)	72 (65–79.7)	87 (80–99)	<0.001
Current Smoking (*n*, %)	55 (13.8%)	15 (9.9%)	24 (16.2%)	16 (16.2%)	0.21
Waist Circumference (cm, mean ± SD)	89.6 ± 13.7	79 ± 8.4	90.6 ± 8.8	103.9 ± 12.3	<0.001
Waist-to-Hip Ratio (mean ± SD)	0.9 ± 0.1	0.8 ± 0.1	0.9 ± 0.1	0.9 ± 0.1	<0.001
BIA Obesity (*n*, %) *	248 (62.9%)	44 (29.3%)	109 (75.2%)	95 (96%)	<0.001
Male	92 (62.6%)	13 (25.5%)	40 (74.1%)	39 (92.9%)	
Female	156 (63.2%)	31 (31.3%)	69 (75.8%)	56 (98.2%)	
DEXA Obesity (*n*, %) *	332 (86%)	95 (65.1%)	141 (97.9%)	96 (100%)	<0.001
Male	123 (85.4%)	30 (61.2%)	52 (96.3%)	41 (100%)	
Female	209 (86.4%)	65 (67%)	89 (98.9%)	55 (100%)	
FMI Obesity (*n*, %) **	130 (33.7%)	2 (1.4%)	39 (27.1%)	89 (92.7%)	<0.001
Male	65 (45.1%)	1 (2%)	25 (46.3%)	39 (95.1%)	
Female	65 (26.9%)	1 (1%)	14 (15.6%)	50 (90.9%)	
Systolic Blood Pressure (mmHg, median (IQR))	116 (110–124)	113 (107.5–120)	118 (112–124)	120 (112.5–127.5)	<0.001
Diastolic Blood Pressure (mmHg, median (IQR))	73 (68.5–77)	71 (66–75)	74 (70–77)	74 (70.5–79.5)	<0.001
Comorbidities (*n*, %)					
Hypertension	46 (11.5%)	13 (8.6%)	16 (10.7%)	17 (17.2%)	0.11
Diabetes	43 (10.8%)	9 (6%)	19 (12.8%)	15 (15.2%)	0.04
Dyslipidemia	36 (9%)	11 (7.3%)	13 (8.7%)	12 (12.1%)	0.42
Cardiovascular Disease	2 (0.5%)	0 (0%)	1 (0.7%)	1 (1%)	0.53
Stroke	1 (0.3%)	0 (0%)	1 (0.7%)	0 (0%)	0.62
Renal Disease	0 (0%)	0 (0%)	0 (0%)	0 (0%)	
Osteoporosis	0 (0%)	0 (0%)	0 (0%)	0 (0%)	
Osteopenia	29 (7.5%)	9 (6.2%)	9 (6.2%)	11 (11.5%)	0.24
Other	58 (14.5%)	19 (12.6%)	27 (18.1%)	12 (12.1%)	0.29

Data presented as median (IQR) for non-normally distributed continuous variables, mean ± SD for normally distributed continuous variables, and *n* (%) for categorical variables. *p*-values calculated using Kruskal–Wallis test for continuous variables and chi-square test or Fisher’s exact test for categorical variables. * Obesity defined as body fat percentage ≥ 25% for males and ≥35% for females for both BIA and DEXA. DEXA data available for 386 participants (13 missing); BIA data available for 394 participants (five missing). FMI data available for 386 participants (13 missing). ** FMI cutpoints (kg/m^2^): men 3–6 normal, >6–9 overfat, >9 obesity; women 5–9 normal, >9–13 overfat, >13 obesity [25]. Age group data were available for 398 participants (one missing). Percentages for variable-specific categorical rows in Table 1 are based on participants with non-missing data for that variable. In Table 1, osteoporosis and osteopenia were study-defined from z-score thresholds: osteoporosis z-score ≤ −2.5; osteopenia z-score > −2.5 to <−1.0. Abbreviations: BMI, body mass index; IQR, interquartile range; SD, standard deviation; DEXA, dual-energy X-ray absorptiometry; BIA, bioelectrical impedance analysis (InBody 770); FMI, fat mass index.

**Table 2:** Cross-classification of index tests against DEXA-derived reference standards (overall sample).

Measure	Reference Positive	Reference Negative	Total
BIA positive	240 (62.2%)	3 (0.8%)	243 (63%)
BIA negative	92 (23.8%)	51 (13.2%)	143 (37%)
Total	332 (86%)	54 (14%)	386 (100%)
BMI ≥ 30 positive (DEXA BF%)	96 (24.9%)	0 (0%)	96 (24.9%)
BMI ≥ 30 negative (DEXA BF%)	236 (61.1%)	54 (14%)	290 (75.1%)
Total	332 (86%)	54 (14%)	386 (100%)
BMI ≥ 30 positive (FMI)	89 (23.1%)	7 (1.8%)	96 (24.9%)
BMI ≥ 30 negative (FMI)	41 (10.6%)	249 (64.5%)	290 (75.1%)
Total	130 (33.7%)	256 (66.3%)	386 (100%)
WHR positive	176 (45.8%)	3 (0.8%)	179 (46.6%)
WHR negative	150 (39.1%)	55 (14.3%)	205 (53.4%)
Total	326 (84.9%)	58 (15.1%)	384 (100%)

DEXA obesity: BF% ≥ 25% males/≥35% females. BIA obesity: same thresholds. WHR: >0.90 males/>0.85 females. DEXA central obesity: A/G ratio >1.0 males/>0.8 females. BIA was cross-classified against DEXA body fat percentage; BMI ≥ 30 was cross-classified against both DEXA body fat percentage and FMI-defined obesity (DEXA-derived); and WHR was cross-classified against DEXA android-to-gynoid ratio. BMI was additionally referenced to FMI because FMI is a height-adjusted fat-mass index and is more directly comparable to BMI. Percentages are calculated using the complete-case denominator for each comparison. Abbreviations: DEXA, dual-energy X-ray absorptiometry; BF%, body fat percentage; BMI, body mass index; WHR, waist-to-hip ratio; A/G ratio, android-to-gynoid fat ratio; BIA, bioelectrical impedance analysis.

**Table 3:** Diagnostic accuracy of index tests against DEXA reference standards, by sex.

Sex	Sensitivity (95% CI)	Specificity (95% CI)	PPV (95% CI)	NPV (95% CI)	LR+	LR−	AUC (95% CI)
BIA BF% vs. DEXA BF%							
Male	73.2 (64.4–80.8)	100.0 (83.9–100.0)	100.0 (96.0–100.0)	38.9 (25.9–53.1)	NA	0.27	0.97 (0.94–0.99)
Female	71.8 (65.1–77.8)	90.9 (75.7–98.1)	98.1 (94.4–99.6)	33.7 (24.0–44.5)	7.89	0.31	0.85 (0.78–0.92)
Total	72.3 (67.1–77.0)	94.4 (84.6–98.8)	98.8 (96.4–99.7)	35.7 (27.8–44.1)	13.01	0.29	0.86 (0.81–0.91)
BMI ≥ 30 vs. DEXA BF%							
Male	33.3 (25.1–42.4)	100.0 (83.9–100.0)	100.0 (91.4–100.0)	20.4 (13.1–29.5)	NA	0.67	0.86 (0.79–0.94)
Female	26.3 (20.5–32.8)	100.0 (89.4–100.0)	100.0 (93.5–100.0)	17.6 (12.5–23.9)	NA	0.74	0.90 (0.85–0.95)
Total	28.9 (24.1–34.1)	100.0 (93.4–100.0)	100.0 (96.2–100.0)	18.6 (14.3–23.6)	NA	0.71	0.89 (0.84–0.93)
BMI ≥ 30 vs. FMI-defined obesity (DEXA-derived)							
Male	60.0 (47.1–72.0)	97.5 (91.2–99.7)	95.1 (83.5–99.4)	74.8 (65.2–82.8)	23.70	0.41	0.95 (0.91–0.98)
Female	76.9 (64.8–86.5)	97.2 (93.5–99.1)	90.9 (80.0–97.0)	92.0 (87.1–95.4)	27.23	0.24	0.96 (0.93–0.99)
Total	68.5 (59.7–76.3)	97.3 (94.4–98.9)	92.7 (85.6–97.0)	85.9 (81.3–89.7)	25.04	0.32	0.95 (0.92–0.97)
WHR vs. DEXA android-to-gynoid ratio							
Male	55.2 (45.7–64.4)	96.3 (81.0–99.9)	98.5 (91.7–100.0)	33.3 (23.1–44.9)	14.90	0.47	0.88 (0.81–0.94)
Female	53.3 (46.3–60.2)	93.5 (78.6–99.2)	98.2 (93.8–99.8)	22.8 (15.9–31.1)	8.27	0.50	0.82 (0.74–0.89)
Total	54.0 (48.4–59.5)	94.8 (85.6–98.9)	98.3 (95.2–99.7)	26.8 (20.9–33.4)	10.44	0.49	0.80 (0.75–0.86)

Sensitivity, specificity, PPV, and NPV presented as percentage (95% CI). AUC derived from receiver operating characteristic curve analysis (95% CI). LR+ = positive likelihood ratio; LR− = negative likelihood ratio. NA = not applicable (specificity 100%, LR+ undefined). Panel A: BIA obesity defined as BF% ≥25% males/≥35% females; DEXA obesity: same thresholds. Overall *n* = 386, males *n* = 144, females *n* = 242. Panel B: FMI-defined obesity: >9 kg/m^2^ males/>13 kg/m^2^ females. Overall *n* = 386, males *n* = 144, females *n* = 242. Panel C: WHR central obesity: >0.90 males/>0.85 females. DEXA central obesity: A/G ratio >1.0 males/>0.8 females. Overall *n* = 384, males *n* = 143, females *n* = 241. Abbreviations: PPV, positive predictive value; NPV, negative predictive value; LR, likelihood ratio; AUC, area under the receiver operating characteristic curve; CI, confidence interval; BF%, body fat percentage; FMI, fat mass index; BMI, body mass index; WHR, waist-to-hip ratio; A/G, android-to-gynoid ratio; DEXA, dual-energy X-ray absorptiometry; BIA, bioelectrical impedance analysis.

**Table 4:** Spearman correlation coefficients with DEXA reference standards, by sex.

Measure	Male	Female	Total	*n* (Total)
Correlated with DEXA-derived measures				
BIA total body fat (%)	0.90	0.75	0.82	386
BMI vs. DEXA body fat (%)	0.77	0.76	0.60	386
BMI vs. FMI (kg/m^2^)	0.87	0.92	0.84	386
Correlated with DEXA android-to-gynoid ratio				
WHR	0.56	0.65	0.69	384

Male *n* = 144 (BIA/DEXA BF%), *n* = 143 (WHR/A/G). Female *n* = 242 (BIA/DEXA BF%), *n* = 241 (WHR/A/G). Abbreviations: FMI, fat mass index; BMI, body mass index; WHR, waist-to-hip ratio; DEXA, dual-energy X-ray absorptiometry; A/G, android-to-gynoid ratio BIA; bioelectrical impedance analysis.

## Data Availability

The datasets used and analysed during the current study are available from the corresponding author on reasonable request.

## Supplementary Materials

The following supporting information can be downloaded at https://www.mdpi.com/article/10.3390/diagnostics16152457/s1, Table S1: STARD 2015 checklist; Table S2: Cross-classification of index tests against DEXA-derived reference standards, by sex; Table S3: FMI category distribution by BMI group; Figure S1: STARD 2015 flow diagram.

## Abbreviations

A/G: Android-to-gynoid (ratio); AUC: Area under the receiver operating characteristic curve; BF%: Body fat percentage; BIA: Bioelectrical impedance analysis; BMI: Body mass index; CI: Confidence interval; DEXA: Dual-energy X-ray absorptiometry; EASO: European Association for the Study of Obesity; FMI: Fat mass index; IQR: Interquartile range; LR: Likelihood ratio; NHANES: National Health and Nutrition Examination Survey; NPV: Negative predictive value; PPV: Positive predictive value; REDCap: Research Electronic Data Capture; ROC: Receiver operating characteristic; SD: Standard deviation; STARD: Standards for Reporting Diagnostic Accuracy Studies; WHO: World Health Organization; WHR: Waist-to-hip ratio.
