# Supplementary material for: Diagnostic Accuracy of Body Mass Index, Bioelectrical Impedance Analysis, and Waist-to-Hip Ratio Against DEXA for Obesity in Saudi Arabia: A Cross-Sectional Study
Source: Diagnostics (Basel). 2026 Aug 4;16(15):2457. doi: 10.3390/diagnostics16152457 (PMC13464004; doi:10.3390/diagnostics16152457)
Supplement: Supplementary file 1 [file diagnostics-16-02457-s001.zip › diagnostics-4459243-supplementary.pdf]

# Diagnostic Accuracy of Body Mass Index, Bioelectrical Impedance Analysis, and Waist-to-Hip Ratio Against DEXA for Obesity in Saudi Arabia: A Cross-Sectional Study


**Authors**

Haifa F. Alotaibi [1], Saleh A. Alqahtani [2,3,4], Alanoud Alfraidi [1], Jamala Selan [1], Feras Altunusi [1], Abdulrahman Albatly [5], Shadan AlMuhaidib [1], Hanan Taib [6], Amr Arafat [1,7] and Waleed Alhazzani [1,8]

**Affiliations**

[1] Research and Innovation Institute, Ministry of Defense Health Services, Riyadh 12426, Saudi Arabia

[2] Liver, Digestive, and Lifestyle Health Research Section, King Faisal Specialist Hospital and Research Center, Riyadh 12713, Saudi Arabia

[3] Organ Transplant Center of Excellence, King Faisal Specialist Hospital and Research Center, Riyadh 12713, Saudi Arabia

[4] Division of Gastroenterology & Hepatology, Weill Cornell Medicine, New York, NY 10065, USA

[5] Department of Nuclear Medicine, Prince Sultan Military Medical City, Ministry of Defense Health Services, Riyadh 12426, Saudi Arabia

[6] Department of Family and Community Medicine, Prince Sultan Military Medical City, Ministry of Defense Health Services, Riyadh 12426, Saudi Arabia

[7] Prince Sultan Cardiac Center, Ministry of Defense Health Services, Riyadh 12426, Saudi Arabia

[8] Critical Care and Internal Medicine Department, College of Medicine, Imam Abdulrahman Bin Faisal University, Dammam 31441, Saudi Arabia

# Table of Contents

SUPPLEMENTARY TABLE S1 STARD 2015 CHECKLIST .......... 3
SUPPLEMENTARY TABLE S2. CROSS-CLASSIFICATION OF INDEX TESTS AGAINST DEXA-DERIVED REFERENCE STANDARDS, BY SEX .......... 6
SUPPLEMENTARY TABLE S3. FMI CATEGORY DISTRIBUTION BY BMI GROUP .......... 8
SUPPLEMENTARY FIGURE S1. STARD 2015 FLOW DIAGRAM .......... 9

**Supplementary Table S1.** STARD 2015 Checklist

| | | | | |
|---|---|---|---|---|
| TITLE / ABSTRACT / KEYWORDS | | | | |
| **1a** | Title | Identification as a study of diagnostic accuracy using at least one measure of accuracy (such as sensitivity, specificity, predictive values, or AUC) | Yes | [p1] Title: "Diagnostic accuracy" stated; Objectives: sensitivity, specificity, PPV, NPV, AUC listed |
| **1b** | Abstract | Structured abstract with study design, methods, results, and conclusions | Yes | Abstract present in current manuscript draft |
| INTRODUCTION | | | | |
| **2** | Introduction | Scientific and clinical background, including the intended use and clinical role of the index test | Yes | [p2] Introduction: Paragraphs 1–4 describe BMI limitations, BIA/DEXA methods, clinical role of each test |
| **3** | Introduction | Study objectives and hypotheses | Yes | [p3] Objectives section: Three primary objectives and secondary objectives explicitly stated |
| METHODS — Study Design | | | | |
| **4** | Methods | Whether data collection was planned before the index test and reference standard were performed (prospective study) or after (retrospective study) | Yes | [p3] Study Design: "We conducted a comparative cross-sectional diagnostic accuracy study." Data Collection: "Each participant underwent all measurements on a single day" — prospective |
| **5** | Methods | Study design: e.g., cross-sectional or cohort study | Yes | [p3] Study Design: "comparative cross-sectional diagnostic accuracy study" |
| METHODS — Participants | | | | |
| **6** | Methods | Eligibility criteria | Yes | [p4] Setting and Participants: aged 18–65 years, males and females. Excluded: pregnant women, metal implants, unable to undergo DEXA |
| **7** | Methods | On what basis potentially eligible participants were identified (such as symptoms, results from previous tests, inclusion in registry) | Yes | [p4] Setting and Participants: "from the waiting area of the primary health care clinic and outpatient clinics at PSMMC, Riyadh" |
| **8** | Methods | Where and when potentially eligible participants were identified (setting, location, and dates) | Yes | [p4] Setting: PSMMC, Riyadh, Saudi Arabia. Dates: "between 14th of October 2025and 17th of February 2026" |
| **9** | Methods | Whether participants formed a consecutive, random, or convenience series | Yes | [p4] Setting and Participants: "Recruitment followed a convenience sampling strategy" |
| METHODS — Test Methods | | | | |
| **10a** | Methods | Index test, in sufficient detail to allow replication | Yes | [p4] Measurement section: InBody 770, multi-frequency (1, 5, 50, 250, 500, 1000 kHz), eight tactile electrodes, barefoot. Anthropometric: BMI formula, WHR measurement sites described |
| **10b** | Methods | Reference standard, in sufficient detail to allow replication | Yes | [p4] Measurement section: Lunar iDXA (GE), Software Version 17, |

| | | | | |
|---|---|---|---|---|
| | | | | trained technician, patient gown, supine, measurements listed |
| **11** | Methods | Rationale for choosing the reference standard (if alternatives exist) | Yes | [p2] Introduction: DEXA described as "the reference standard for body composition analysis" [14] |
| **12a** | Methods | Definition of and rationale for test positivity cut-offs or result categories of the index test, distinguishing pre-specified from exploratory | Yes | [p5] Definitions section: BIA obesity ≥25% M/≥35% F [23]; BMI ≥30 [1]; WHR >0.90 M/>0.85 F [25]; all pre-specified with citations |
| **12b** | Methods | Definition of and rationale for test positivity cut-offs or result categories of the reference standard, distinguishing pre-specified from exploratory | Yes | [p5] Definitions section: DEXA BF% ≥25% M/≥35% F [23]; FMI categories with sex-specific thresholds [24]; A/G ratio >1.0 M/>0.8 F [26]; all pre-specified |
| **13a** | Methods | Whether clinical information and reference standard results were available to the performers/readers of the index test | Yes | [p4] Measurement section: Results extracted after completion of all assessments; neither measurement subject to reader interpretation |
| **13b** | Methods | Whether clinical information and index test results were available to the assessors of the reference standard | Yes | [p5] Index test and reference standard results were not available to the respective operators at the time of measurement, as results were extracted after completion of all assessments. |
| METHODS — Analysis | | | | |
| **14** | Methods | Methods for estimating or comparing measures of diagnostic accuracy | Yes | [p5] Statistical Analysis: 2×2 tables, sensitivity, specificity, PPV, NPV, LR+, LR−, ROC curves, AUC, 95% CI, Spearman correlations. Overall and stratified by sex |
| **15** | Methods | How indeterminate index test or reference standard results were handled | Yes | [p5] Statistical Analysis: "complete-case analysis." Missing data reported: DEXA 386/399 (13 missing), BIA 394/399 (5 missing), FMI 386/399 (13 missing) |
| **16** | Methods | How missing data on the index test and reference standard were handled | Yes | [p5] Same as item 15: complete-case analysis with explicit counts of available data per comparison |
| **17** | Methods | Any analyses of variability in diagnostic accuracy, distinguishing pre-specified from exploratory | Yes | [p5] Statistical Analysis: stratification by sex pre-specified in objectives. BMI category stratification stated in Sample Size section |
| **18** | Methods | Intended sample size and how it was determined | Yes | [p4] Sample Size: Buderer's formula, 90% sensitivity, 85% specificity, 20% prevalence, 0.07 precision, 95% CI. Required n=353, target with 10% dropout = 393 |
| METHODS — Ethics | | | | |
| **19** | Methods | Ethics approval and informed consent | Yes | [p3] Ethical Considerations: Written informed consent. Central Ethics Review Board at MODHS (approval 11-2025-02-03-108). REDCap storage, no personal identifiers |

| | | | | |
|---|---|---|---|---|
| RESULTS — Participants | | | | |
| **20** | Results | Flow of participants, using a diagram (recommended) | Yes | Flow Diagram Included |
| **21a** | Results | Baseline demographic characteristics of participants | Yes | [p6] Table 1: age, sex, height, weight, smoking, waist circumference, WHR, blood pressure, comorbidities, stratified by BMI group |
| **21b** | Results | Clinical characteristics of participants | Yes | [p6] Table 1: hypertension, diabetes, dyslipidaemia, cardiovascular disease, stroke, renal disease, other comorbidities |
| **22** | Results | Existing diagnoses and comorbidity at the time of the study | Yes | [p6] Participant Characteristics: Diabetes (p=0.04), hypertension (p=0.11), dyslipidaemia, CVD, stroke reported across BMI groups |
| RESULTS — Test Results | | | | |
| **23** | Results | Time interval and any clinical interventions between index test and reference standard | Yes | [p5] Directly from one measurement to the next with no fixed time interval and no clinical intervention between the index tests and the reference standard |
| **24** | Results | Cross tabulation of the index test results (or their distribution) by the results of the reference standard | Yes | Supplementary Table S2 cross-classification tables for each comparison, overall and by sex |
| **25** | Results | Estimates of diagnostic accuracy and their precision (such as 95% confidence intervals) | Yes | [p10] Table 3: Diagnostic accuracy table. Sensitivity, specificity, PPV, NPV, LR+, LR−, AUC with 95% CI for BIA vs DEXA, BMI vs FMI, WHR vs A/G ratio; overall and by sex |
| **26** | Results | Any adverse events from performing the index test or the reference standard | Yes | No adverse events from InBody or DEXA procedures |
| **27** | Results | Table of all study results, including indeterminate and missing results | Yes | [p6-12] Tables 1,2,3,4 diagnostic accuracy summary tables, Spearman correlation tables. Missing data counts stated in Statistical Analysis |
| DISCUSSION | | | | |
| **28** | Discussion | Study limitations, including sources of potential bias, statistical uncertainty, and generalisability | Yes | [p14] Limitations paragraph: convenience sampling, military hospital setting, non-standardized fasting, cross-sectional design, 6% sample shortfall, no ethnicity data beyond nationality |
| **29** | Discussion | Implications for practice, including the intended use and clinical role of the index test | Yes | [p15] Clinical and Public Health Implications paragraph: BMI underestimation, BIA as intermediate tool, WHR limitations, resource allocation implications |
| OTHER INFORMATION | | | | |
| **30** | Other | Registration number and name of registry | N/A | Prospective registration is not mandated for observational studies |

| | | | | |
|---|---|---|---|---|
| **31** | Other | Where the full study protocol can be accessed | Yes | The study protocol is available from the corresponding author upon reasonable request |
| **32** | Other | Sources of funding and other support; role of funders | N/A | [p15] The study receive no funding |

Reference: Cohen JF, Korevaar DA, Altman DG, et al. STARD 2015 guidelines for reporting diagnostic accuracy studies: explanation and elaboration. BMJ Open. 2016;6(11):e012799.

**Supplementary Table S2.** Cross-classification of index tests against DEXA-derived reference standards, by sex

| **Sex** | **Test result** | **Reference positive** | **Reference negative** | **Total** |
|---|---|---|---|---|
| **BIABF% vs DEXA BF%** | | | | |
| **Male** | Positive | 90 (62.5%) | 0 (0%) | 90 (62.5%) |
| | Negative | 33 (22.9%) | 21 (14.6%) | 54 (37.5%) |
| | Total | 123 (85.4%) | 21 (14.6%) | 144 (100%) |
| **Female** | Positive | 150 (62%) | 3 (1.2%) | 153 (63.2%) |
| | Negative | 59 (24.4%) | 30 (12.4%) | 89 (36.8%) |
| | Total | 209 (86.4%) | 33 (13.6%) | 242 (100%) |
| **BMI ≥30 vs DEXA BF%** | | | | |
| **Male** | Positive | 41 (28.5%) | 0 (0%) | 41 (28.5%) |
| | Negative | 82 (56.9%) | 21 (14.6%) | 103 (71.5%) |
| | Total | 123 (85.4%) | 21 (14.6%) | 144 (100%) |
| **Female** | Positive | 55 (22.7%) | 0 (0%) | 55 (22.7%) |
| | Negative | 154 (63.6%) | 33 (13.6%) | 187 (77.3%) |
| | Total | 209 (86.4%) | 33 (13.6%) | 242 (100%) |
| **BMI ≥30 vs FMI-defined obesity (DEXA-derived)** | | | | |
| **Male** | Positive | 39 (27.1%) | 2 (1.4%) | 41 (28.5%) |
| | Negative | 26 (18.1%) | 77 (53.5%) | 103 (71.5%) |
| | Total | 65 (45.1%) | 79 (54.9%) | 144 (100%) |

| Sex | Test result | Reference positive | Reference negative | Total |
|---|---|---|---|---|
| **Female** | Positive | 50 (20.7%) | 5 (2.1%) | 55 (22.7%) |
| | Negative | 15 (6.2%) | 172 (71.1%) | 187 (77.3%) |
| | Total | 65 (26.9%) | 177 (73.1%) | 242 (100%) |
| **WHR vs DEXA android-to-gynoid ratio** | | | | |
| **Male** | Positive | 64 (44.8%) | 1 (0.7%) | 65 (45.5%) |
| | Negative | 52 (36.4%) | 26 (18.2%) | 78 (54.5%) |
| | Total | 116 (81.1%) | 27 (18.9%) | 143 (100%) |
| **Female** | Positive | 112 (46.5%) | 2 (0.8%) | 114 (47.3%) |
| | Negative | 98 (40.7%) | 29 (12%) | 127 (52.7%) |
| | Total | 210 (87.1%) | 31 (12.9%) | 241 (100%) |

BIA was cross-classified against DEXA body fat percentage; BMI ≥30 was cross-classified against both DEXA body fat percentage and FMI-defined obesity (DEXA-derived); and WHR was cross-classified against DEXA android-to-gynoid ratio.

Abbreviations: DEXA, dual-energy X-ray absorptiometry; BF%, body fat percentage; BMI, body mass index; FMI, fat mass index; WHR, waist-to-hip ratio.

DEXA obesity: BF% ≥25% males / ≥35% females. BIA obesity: same thresholds. BMI obesity: ≥30 kg/m². WHR: >0.90 males / >0.85 females. DEXA central obesity: A/G ratio >1.0 males / >0.8 females.

***Abbreviations:*** DEXA, dual-energy X-ray absorptiometry; BF%, body fat percentage; BMI, body mass index; WHR, waist-to-hip ratio; A/G ratio, android-to-gynoid fat ratio.

**Supplementary Table S3.** FMI category distribution by BMI group

| FMI category | Total | BMI ≤24.9 | BMI 25.0-29.9 | BMI ≥30.0 | p-value |
|---|---|---|---|---|---|
| Normal | 102 (26.4%) | 97 (66.4%) | 5 (3.5%) | 0 (0%) | < 0.001 |
| Overfat | 154 (39.9%) | 47 (32.2%) | 100 (69.4%) | 7 (7.3%) | |
| Obese | 130 (33.7%) | 2 (1.4%) | 39 (27.1%) | 89 (92.7%) | |

FMI cut-points ($kg/m^2$): men 3–6 normal, >6–9 overfat, >9 obese; women 5–9 normal, >9–13 overfat, >13 obese.

Abbreviations: FMI, fat mass index; BMI, body mass index.

**Supplementary Figure S1.** STARD 2015 Flow Diagram

**Potentially eligible participants**
Adults aged 18–65 years approached in outpatient clinics
at Prince Sultan Military Medical City, Riyadh, Saudi Arabia
*October 2025 – February 2026*

**Enrolled participants**
Provided written informed consent
**(n = 399)**

**Excluded prior to enrollment**

**Exclusion criteria:**
- Pregnant women
- Metal implants
- Unable to undergo DEXA scanning

**All index tests and reference standard performed**

**Index tests:** InBody 770 BIA (body fat %), BMI (≥30 kg/m²), WHR (>0.90 M / >0.85 F)
**Reference standard:** DEXA (Lunar iDXA, GE) — body fat %, FMI, android-to-gynoid ratio
**(n = 399)**

**Participants excluded from analysis due to missing data**

**DEXA data missing:** 13 participants (386 with complete DEXA data)
**BIA data missing:** 5 participants (394 with complete BIA data)
**WHR or DEXA A/G data missing:** 15 participants (384 with complete WHR + A/G data)
**FMI (DEXA-derived) missing:** 13 participants (386 with complete FMI data)

**Diagnostic accuracy analyses (complete-case)**

**Comparison 1: BIA BF% vs DEXA BF%**
*Obesity: BF% ≥25% males / ≥35% females*
n = 386 (males 144, females 242)

Index test + / Reference +: 240 (TP)
Index test + / Reference –: 3 (FP)
Index test – / Reference +: 92 (FN)
Index test – / Reference –: 51 (TN)

**Comparison 2: BMI ≥30 vs DEXA BF%**
*Obesity: BF% ≥25% males / ≥35% females*
n = 386 (males 144, females 242)

Index test + / Reference +: 96 (TP)
Index test + / Reference –: 0 (FP)
Index test – / Reference +: 236 (FN)
Index test – / Reference –: 54 (TN)

**Comparison 3: BMI ≥30 vs FMI (DEXA-derived)**
*FMI obesity: >9 males / >13 females kg/m²*
**n = 386** (males 144, females 242)

Index test + / Reference +: 89 (TP)
Index test + / Reference –: 7 (FP)
Index test – / Reference +: 41 (FN)
Index test – / Reference –: 249 (TN)

**Comparison 4: WHR vs DEXA android/gynoid ratio**
*WHR: >0.90 males / >0.85 females*
**n = 384** (males 143, females 241)

Index test + / Reference +: 176 (TP)
Index test + / Reference –: 3 (FP)
Index test – / Reference +: 150 (FN)
Index test – / Reference –: 55 (TN)

STARD 2015 flow diagram showing participant flow through the COMPARE-DEXA diagnostic accuracy study. All participants underwent index tests (InBody 770 bioelectrical impedance analysis, BMI, waist-to-hip ratio) and the reference standard (dual-energy X-ray absorptiometry) on the same day. Four diagnostic comparisons were performed using complete-case analysis. Cross-classification results are presented as true positive (TP), false positive (FP), false negative (FN), and true negative (TN) counts.

***Abbreviations***: BF%, body fat percentage; BMI, body mass index; DEXA, dual-energy X-ray absorptiometry; FMI, fat mass index; FN, false negative; FP, false positive; TN, true negative; TP, true positive; WHR, waist-to-hip ratio; A/G, android-to-gynoid ratio; BIA, bioelectrical impedance analysis.
